# An *In Vitro* and *In Vivo* Comparison of Osteogenic Differentiation of Human Mesenchymal Stromal/Stem Cells

**DOI:** 10.1155/2021/9919361

**Published:** 2021-09-08

**Authors:** Jamie Mollentze, Chrisna Durandt, Michael S. Pepper

**Affiliations:** Institute for Cellular and Molecular Medicine, Department of Immunology; SAMRC Extramural Unit for Stem Cell Research and Therapy, Faculty of Health Sciences, University of Pretoria, Pretoria, South Africa

## Abstract

The use of stem cells in regenerative medicine, including tissue engineering and transplantation, has generated a great deal of enthusiasm. Mesenchymal stromal/stem cells (MSCs) can be isolated from various tissues, most commonly, bone marrow but more recently adipose tissue, dental pulp, and Wharton's jelly, to name a few. MSCs display varying phenotypic profiles and osteogenic differentiating capacity depending and their site of origin. MSCs have been successfully differentiated into osteoblasts both *in vitro* an *in vivo* but discrepancies exist when the two are compared: what happens *in vitro* does not necessarily happen *in vivo*, and it is therefore important to understand why these differences occur. The osteogenic process is a complex network of transcription factors, stimulators, inhibitors, proteins, etc., and *in vivo* experiments are helpful in evaluating the various aspects of this osteogenic process without distractions and confounding variables. With that in mind, the results of *in vitro* experiments need to be carefully considered and interpreted with caution as they do not perfectly replicate the conditions found within living organisms. This is where *in vivo* experiments help us better understand interactions that might occur in the osteogenic process that cannot be replicated *in vitro*. Potentially, these differences could also be exploited to develop an optimal MSC cell therapeutic product that can be used for bone disorders. There are many bone disorders, most of which cause a great deal of discomfort. Clinically acceptable protocols could be developed in which MSCs are used to aid in bone regeneration providing relief for patients with chronic pain. The aim of this review is to examine the differences between studies conducted *in vitro* and *in vivo* with regard to the osteogenic process to better define the gaps in current osteogenic research. By better understanding osteogenic differentiation, we can better define treatment strategies for various bone disorders.

## 1. Introduction

*In vitro* experiments have increased in complexity over the past several years from the use of omic technologies to study the cellular activity of primary cells to immortalized cells that overexpress telomerase allowing one to create human cell lines with normal or abnormal phenotypes. They have been also allowed for the use of stromal/stem cells that have the ability to differentiate into various tissues under the influence of diverse stimuli outside the living organism. There have also been advances in bioengineering/material science that have allowed for the development of *in vitro* multiorgan systems. All of these *in vitro* developments have allowed us to better understand the mechanisms of how cells operate under set experimental conditions at relatively low cost. These *in vitro* models have helped us to study a wide range of diseases and have provided the basis for many treatment strategies. However, many of these treatment strategies have also fallen short when tested in animal/human studies, as *in vitro* studies isolate specific process which do not represent what is truly happening within an organism. *In vivo* models should bridge this gap. For an *in vivo* model to be successful, it should reflect the physiology and biomechanics of certain aspects of what happens within the human body.

True stem cells are defined as undifferentiated cells which simultaneously possess self-renewal and differentiation ability. Their capacity to differentiate into several cell types has solicited a great deal of interest in the fields of cell- and gene-based therapy, and regenerative medicine [1]. Langer and Vacanti [2] defined tissue engineering as “an interdisciplinary field that applies the principles of engineering and life sciences toward the development of biological substitutes that restore, maintain, or improve tissue function”. Regenerative medicine, therefore, largely depends on the ability of stem cells to differentiate into the cell type of interest, replacing damaged or dysfunctional cells at the site of injury, and in so doing, restoring structure and function to the damaged tissue or organ [3]. The development of tissue engineering that specifically focusses on bone regeneration is important for bony defects and also when a fractured bone does not heal resulting in a nonunion [4]. Bone regeneration is a lengthy and complicated process, and orthopaedic surgeons often face bone regeneration that is suboptimal. The ability of mesenchymal stromal/stem cells (MSCs) to differentiate into osteoblasts has prompted surgeons and researchers to investigate the use of MSCs, in combination with a biomaterial scaffold, to improve bone repair and regeneration [5]. The use of MSCs *in vitro* to study the osteogenic process can help simplify this complex process and help us study individual processes that occur throughout osteogenesis. However, *in vivo* osteogenic experiments are equally if not more important to understand the osteogenic process as a whole.

## 2. Osteogenesis

Osteogenesis can be divided into intramembranous and endochondral ossification processes. Intramembranous ossification occurs in the craniofacial bones and clavicle and involves the direct differentiation of MSCs into osteocytes to form bone, while endochondral ossification involves the differentiation of MSCs into chondrocytes to form cartilage, which then forms a template for bone formation. Endochondral ossification is responsible for the formation of the long, short, and irregular bones that form part of the axial and appendicular skeleton [6].

**Figure 1:**
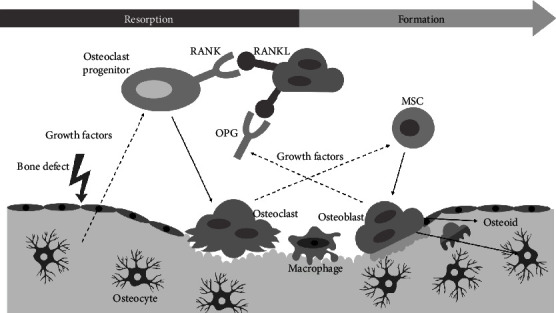
Schematic representation of the bone remodelling process. Solid lines indicate differentiation, and dotted lines indicate stimulation. Osteocytes within bone tissue stimulate osteoclast progenitor cells to differentiates into osteoclasts. Osteoblasts can also stimulate osteoclast progenitor cells through RANK/RANKL binding. Once the defective bone tissue is cleared, macrophage-like cells smooth the resorbed bone tissue. Before undergoing apoptosis, osteoclasts recruit osteoblasts for matrix deposition. Osteoblasts stimulate the release of osteoprotegerin (OPG) that acts as a soluble decoy and inhibits osteoclast differentiation. Adapted from Wittkowske et al. [12], bone remodelling cycle, https://creativecommons.org/licenses/by/4.0/legalcodehttp://creativecommons.org/licenses/by/4.0/. MSC: mesenchymal stroma/stem cell; RANK: receptor activator of nuclear factor kappa-*Β*; RANKL: receptor activator of nuclear factor kappa-*Β* ligand; OPG: osteoprotegerin.

Bone is a highly dynamic tissue and involves the constant build up and breakdown of bone tissue known as bone remodelling. Bone is composed of both cells (osteocytes, osteoblasts, and osteoclasts) and an extracellular matrix that is mineralized by the deposition of calcium hydroxyapatite [7]. Bone matrix homeostasis is monitored and maintained by mature bone cells known as osteocytes [8]. When matrix microdamage occurs, such as in a fracture, disruption of osteocyte canaliculi leads to the paracrine release of cytokines and other mediators by osteocytes, attracting osteoclasts to the site of injury/defect [9, 10]. Osteoclastogenesis (osteoclast differentiation) from mononuclear osteoclast precursors can also be induced by the secretion of receptor activator of nuclear factor kappa-B ligand (RANKL) and macrophage-colony stimulating factor (M-CSF) by surrounding stromal and osteoblast cells [11, 12]. Osteoclasts secrete a collagen-digesting enzyme and an acidic hydrogen ion mixture that dissolves the calcium phosphate in the defective bone tissue, a process known as bone resorption [13]. Once the defective bone tissue is cleaned out, macrophage-like cells smooth the resorbed bone tissue in preparation for matrix deposition [12]. Osteoclasts then recruit bone-forming cells termed osteoblasts before they undergo apoptosis. Osteoblasts are responsible for synthesizing components of the bone matrix, such as type I collagen, proteoglycan, and alkaline phosphatase (ALP), to name a few [7]. By balancing bone resorption and bone formation, bone homeostasis is maintained (Figure 1).

## 3. Regulation of Osteogenesis

Osteogenesis is controlled by a wide range of stimulators and inhibitors, which occur both at the transcriptional level and through extracellular signalling pathways. Runt-related transcription factor 2 (RUNX2) is an essential transcription factor that controls the differentiation of MSCs into osteoblasts [14]. Additionally, osteogenesis is regulated through changes in the osteoprotegerin (OPG)/RANKL ratio. RANKL binds to RANK, found on the surface of preosteoclasts, to induce differentiation of preosteoclasts into mature osteoclasts in the presence of M-CSF, leading to bone resorption [7]. Osteoclast differentiation needs to be blocked in order for osteoblast differentiation to occur; this happens through the secretion of OPG that acts as a soluble decoy receptor, which binds to RANKL, blocking RANKL/RANK interactions and thereby inhibiting osteoclast differentiation (Figure 1).

### 3.1. RUNX2: Master Regulator of Osteogenic Transcription

**Figure 2:**
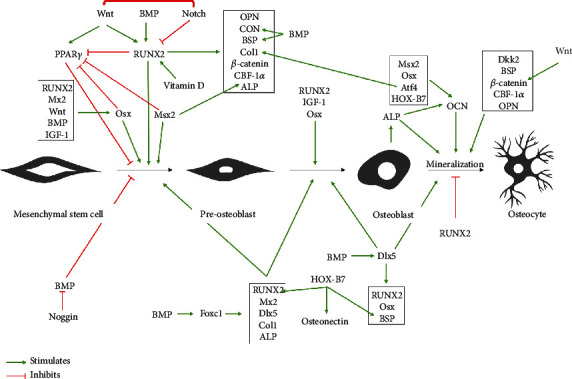
Regulation of MSC osteogenic differentiation. Green arrows indicate positive regulation while red lines indicate negative regulation. This figure illustrates the complex network of cells and mediators involved in bone formation.

RUNX2 is the main molecular regulator responsible for the differentiation of MSCs into preosteoblasts and is expressed early to promote osteogenesis and inhibit adipogenesis and chondrogenesis [15]. RUNX2 regulates many downstream osteogenic genes such as Osterix (Osx), osteocalcin (Ocn), ALP, *β*-catenin, core-binding factor-1*α* (CBF-1*α*), bone sialoprotein (BSP), osteonectin, osteopontin (Opn), and type I collagen, to name a few (Figure 2). Furthermore, activation/overexpression of RUNX2 results in a significant decrease in adipogenic-related transcription factors and enzymes, peroxisome proliferator-activated receptor (PPAR*γ*), and lipoprotein lipase (LPL) [16]. RUNX2 is downregulated during the later stages of bone maturation [15]. *In vitro* studies show that RUNX2 also directly regulates synthesis of both OPG and RANKL [17, 18]. These findings have been confirmed *in vivo* [16, 19]. Otto et al. [19] showed that a mutation in the RUNX2 gene resulted in a complete absence of osteoblasts, which resulted in turn in a cartilaginous skeleton; RUNX2-deficient mice also die shortly after birth. A later study done by Adhami et al. [20] demonstrated that RUNX2 null mice were born alive and were identical to wildtype mice and only after a month did the RUNX2 null mice display poor growth, weighing 20-25% less than their wildtype counterparts. With closer inspection, they found there was a 50% decrease in trabecular number and a 20% decrease in trabecular thickness indicating that the loss of RUNX2 led to significant growth deficits. They also noticed impaired bone mineralization due to a decrease in the average density of hydroxyapatite. Together, these findings indicate that RUNX2 is important in bone formation. The difference between the two studies could be explained by differences in the strain of mice. On the other hand, Zhang et al. [16] demonstrated that the overexpression of RUNX2 in 4-week-old nude mice resulted in increased mineral deposition.

### 3.2. Early-Stage Osteogenic Regulators

Msx2 is a homeobox transcription factor that mainly controls the early stages of osteogenic differentiation but also plays a role in the later stages of osteoblastic mineralization. *Ex vivo* studies have shown that the expression of Msx2 promoted upregulation of Osx and ALP, but did not influence the expression of RUNX2 [21]. Cheng et al. [21] demonstrated the ability of Msx2 to regulate osteogenesis through the suppression PPAR*γ*. Ocn, a late stage osteogenic marker, is downregulated in the early stages of osteogenesis through protein-protein interactions between Msx2 and Ocn [22]. Satokata et al. [23] reported osteoblast deficiency leading to osteoporosis syndromes in Msx2 null mice, supporting the idea that Msx2 plays an important role in osteogenic differentiation. The insulin-like growth factor (IGF) axis regulates both osteoblast and osteoclast differentiation and is one of the most abundant growth factors in bone tissue [24]. Osteocytes upregulate IGF-1 in response to mechanical loading; IGF-1 is thus considered to be an early osteogenic marker [25]. The knockout of IGF-1 in MSCs compromises the osteogenic process *in vitro* [26]. This study was corroborated by Zhang et al. [27] who showed that bone formation was completely blocked by disrupting the *Igf1* gene in mature osteoblasts. Similarly, *in vivo*, a disruption in the *Igf1* gene inhibited periosteal expansion resulting in rodents with smaller body features [28].

Osx and activating transcription factor 4 (Atf4) are located downstream of RUNX2 and are both important transcription factors in osteogenesis. Atf4 regulates osteogenesis through its ability to regulate Ocn and collagen type I. Deletion of Atf4 in mice led to impaired terminal osteoblast differentiation and resulted in severe osteopenia and other defects during skeletal development [29, 30]. Osx is a potent bone forming stimulator that is part of the specificity protein 1 family [31]. Osx stimulates osteoblastic differentiation in MSCs through the repression of PPAR*γ*, which inhibits adipogenesis [32]. Several *in vivo* studies have demonstrated the indispensable function of Osx in osteogenic differentiation [31, 33–35]. The importance of Osx was demonstrated by Hilton et al. [33]: inhibition of Osx impairs osteoblast mineralization of cartilage into bone. *In vitro* studies suggest that Osx is modulated by IGF-I, BMPs, Msx2, and the Wnt signalling pathway [31, 34, 35]. Overexpression of Osx in C2C12 cells resulted in increased expression of ALP and Ocn, leading to the calcification of bone tissue [31]. ALP plays an important role in phosphate metabolism by hydrolysing inorganic phosphate to promote matrix calcification, thus playing a key role during osteogenesis [36]. Nakamura et al. [37] overexpressed ALP in wild-type osteoblast cells which resulted in increased expression of osteogenic genes RUNX2, Osx, Ocn, and dentin matrix acidic phosphoprotein 1 (Dmp1), an osteocyte differentiation marker. Consistent with Nakamura et al.'s [37] *in vitro* study, Narisawa et al. [38] demonstrated that the overexpression of ALP by osteoblasts resulted in an increase in bone mineralization *in vivo*. ALP-/- mice exhibit long bone and skull fusion defects, and by administering exogenous ALP, the authors were able to increase bone density and the life span of these mice [38–42]. Another early-stage osteogenic marker is *COL1A1*. Mutations in *COL1A1* have been studied extensively in osteogenesis imperfecta, a genetic disorder that results in bone fragility and multiple fractures. *COL1A1* is important for the synthesis of collagen type I which is a major component of bone extracellular matrix (ECM) and is expressed in all osteoblastic cells throughout osteogenic differentiation, and mutations lead to ineffective or absent differentiation [43, 44].

### 3.3. Late-Stage Osteogenic Regulators

Transcription factors involved in the later stages of osteogenesis regulate terminal differentiation and are involved in mineralization. Some of the most important late-stage transcription factors are Opn, distal less homeobox 5 (Dlx5), Ocn, OPG, and BSP, to name a few. Opn is a matricellular protein that belongs to the small integrin-binding ligand N-linked glycoprotein (SIBLING) family and is involved in mineralization in response to mechanical stress. Chen et al. [45] observed that Opn-/- MSCs form considerably less bone tissue *in vitro* compared to their wild-type counterparts; however, the same is not true *in vivo*. Chen et al. [45] suggest that the difference between *in vitro* and *in vivo* studies may reflect functional redundancy and that other members of the SIBLING family can compensate for Opn deficiency. Interestingly, however, Opn-/- mice did show a higher fat weight/body weight ratio. Dlx5 is another bone inducing transcription factor that plays a role in the later stages of osteogenesis. *In vitro* studies show that by inhibiting Dlx5, RUNX2 and Osx expression was blocked, suggesting that Dlx5 may be an upstream regulator of RUNX2 and Osx. Dlx5 is also a downstream target of BMP signalling [46]. Additionally, upregulation of Dlx5 did not increase the osteogenic markers ALP and Ocn *in vitro*. Other cell culture studies demonstrated however that overexpression of Dlx5 increases expression of Ocn [47]. Dlx5 null osteoblasts display a higher RANKL/OPG ratio, suggesting that Dlx5-deficient osteoblasts are able to induce osteoclastogenesis [48]. Dlx5-deficient mice displayed delayed and abnormal osteogenesis, resulting in severe craniofacial abnormalities as well as a decrease in RUNX2, Osx, Ocn, and BSP expression [48, 49]. An increase in the number of osteoclasts was observed in the femurs of Dlx5 null mice [50]. Bone defects were also present in Dlx5/Dlx6 double knockout mice, further indicating that Dlx5 plays an important role in bone mineralization [50]. Interestingly, the forced overexpression of Dlx5 *in vivo* also resulted in reduced bone mineralized matrix deposition despite high levels of RUNX2 and BSP expression, suggesting a block in the later stages of osteogenesis [51].

OPG is expressed by osteoblasts, MSCs, and endothelial cells and can enhance osteogenesis by acting as a decoy receptor for RANKL, inhibiting osteoclastogenesis [52, 53]. Both *in vitro* and *in vivo* models have demonstrated that OPG levels are inversely related to osteoclastogenesis [54, 55]. In an *in vitro* study, the treatment of undifferentiated MSCs with OPG resulted in the enhancement of osteogenesis [56]. Furthermore, OPG knockout mice demonstrate an increase in bone resorption due to increased osteoclast activity [57].

Ocn and BSP are both noncollagenous proteins found in bone tissue. Ocn is the most abundant, noncollagenous protein in bone tissue and is used as a biochemical marker for bone formation *in vitro* and *in vivo*: an increase in Ocn levels has been associated with an increase in bone mineral density [58]. BSP is found in mineralized tissue such as bone, calcified cartilage, and dentin and makes up approximately 8% of the noncollagenous protein of bone [59]. Although the function of BSP is not yet fully known, it is suspected to play a role in the formation of hydroxyapatite (essential component of healthy bone tissue) [60]. In the absence of BSP *in vitro*, osteogenic differentiation is negatively impacted. BSP overexpression leads to an increase in osteoblast-related gene expression as well as enhanced mineralization. The opposite is also true; when BSP expression is reduced, there is both a reduction in osteoblast-related gene expression and bone mineralization [61]. *In vitro* studies have suggested that a lack of BSP reduces osteoprogenitor cell numbers and has a compensatory role on Opn. The BSP-/- phenotype is associated with the upregulation of Opn in an attempt to rescue the cells. However, the overexpression of Opn is not enough to rescue the cells, and thus, bone formation and mineralization do not occur [62, 63]. BSP-/- mice demonstrate normal skeletal development; however, they display undermineralization of long bones [63, 64].

### 3.4. Additional Osteogenic Transcription Factors

Other transcription factors that are involved in osteogenic differentiation are frizzled-related protein (FRZB), dickopf (Dkk) 2, homeobox protein Hox-B7 (HOXB7), *β*-catenin, and others. FRZB is a Wnt modulator that increases the expression of osteogenic-related markers and calcium deposition. The overexpression of Frzb in MC3T3-E1 cells increases osteogenic activity while the loss of Frzb results in a decrease in osteogenic activity [65]. However, Frzb null mice show an increase in cortical bone thickness [66]. These contrasting results may be explained by the deficiency of FRZB leading to supraphysiological levels of other Wnt modulators such as Dkk1 and Dkk2 that stimulate osteogenesis. Dkk1 and Dkk2 work antagonistically *in vivo*, where the increased expression of Dkk1 results in a decrease in bone mass while an increase in Dkk2 expression positively stimulates bone formation [67, 68].

When the transcription factor HOXB7 is over expressed, osteogenesis is enhanced through the upregulation of RUNX2 [69]. Gao et al. [69] performed both *in vitro* and *in vivo* studies to investigate the role of HOXB7 during osteogenic differentiation. In their *in vitro* studies, the overexpression of HOXB7 enhanced bone mineralization through activation of ALP. HOXB7 overexpression also had an effect on other osteogenic transcription factors and proteins such as RUNX2, osteonectin, collagen type I, BSP, and Ocn, leading to the promotion of osteogenesis. In contrast, when HOXB7 was inhibited, these transcription factors were downregulated resulting in a decrease in ALP activity that led to a decrease in mineralization. Other HOX genes involved in osteogenesis are HOXa2 and HOXd9. *In vivo* studies showed that during bone regeneration, HOXa2 is upregulated after bone fracture while HOXd9 is downregulated [70].

The *β*-catenin protein is multifunctional. One important function is its ability to regulate the transduction of Wnt signalling [71]. The inhibition of *β*-catenin leads to the inhibition of osteogenesis and the promotion of chondrogenesis [72]. *β*-catenin is activated by the Wnt signalling pathway; *β*-catenin then interacts with LEF/TCF which together increase bone mineralization [73]. *Ex vivo* studies have demonstrated the importance of *β*-catenin in osteoblast mineralization through its downstream regulation of BMP2 [74]. In an *in vivo* study, Hill et al. [75] knocked-down *β*-catenin from head and limb mesenchyme in mouse embryos. In the absence of *β*-catenin, the mutant mice did not form cortical or trabecular bone. Interestingly, the overexpression of *β*-catenin does not result in an increase in osteoblast number, but rather inhibits chondrogenesis and allows for MSC osteogenesis [75].

There are several other transcription factors, not discussed in this review, that are involved in osteogenesis. These include matrix extracellular phosphoglycoprotein (MEPE), human high-temperature requirement protein 1 (HTRA1), IGFBP-2, and secreted protein acidic and rich in cysteine (SPARC), TMEM119, sclerostin, and hypoxia-inducible factor-1*α* (HIF-1*α*); for further information, please refer to [76–83].

The osteogenic process involves a complex network of cells and mediators, and even the slightest disruption of the network leads to defective bone formation (Figure 2).

#### 3.4.1. Signalling Pathways

**Figure 3:**
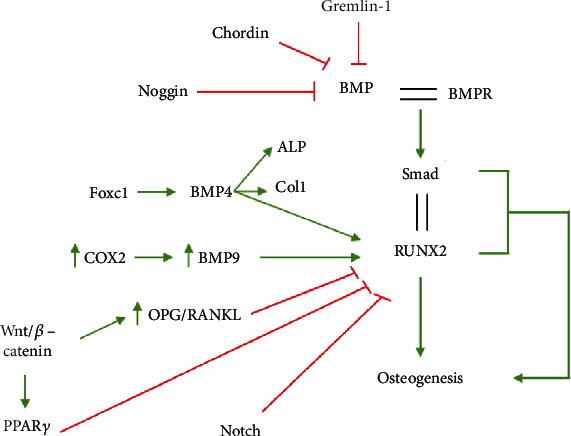
Illustration of how various signalling pathways regulate osteogenesis through the master regulator of osteogenesis, RUNX2. Green arrows indicate positive regulation while red lines indicate negative regulation.

Successful translation of *in vitro* findings to clinical applications *in vivo* requires a good understanding of potential differences in events during *in vitro* and *in vivo* regulation of osteogenic differentiation. The BMP pathway and the Wnt/*β*-catenin signalling pathway are two important extracellular signalling pathways involved in osteogenic differentiation [72, 84]. Several studies have investigated the role of the BMP pathway during *in vitro* and *in vivo* osteogenic differentiation and reported on the differences and similarities in extracellular signalling pathways regulating events in these settings. Tsialogiannis et al. [85] concluded that the BMP pathway plays an important role during both *in vitro* and *in vivo* osteogenic differentiation. The majority of studies looking at the relationship between the Wnt/*β*-catenin signalling pathway and bone formation have been done *in vivo*. Other extracellular signalling pathways that play a role in osteogenesis are the Notch signalling pathway, the hedgehog pathway, fibroblast growth factor (FGF), vascular endothelial growth factor (VEGF), and extracellular signal-regulated kinase [86] (Figure 3).

### 3.5. BMP Signalling Pathway

BMP binds to its receptor, BMPR, found on epithelial cells, which in turn activates the intracellular transcription factor Smad. Smad binds to the master regulator, RUNX2. The Smad-RUNX2 complex induces osteogenesis [87] (Figure 3). Using various BMP antagonists *in vitro*, Tsialogiannis et al. [85] demonstrated that inhibition of BMP function affects multiple downstream factors, such as RUNX2, BSP, and Ocn. The investigators extended their investigation by overexpressing noggin, a BMP antagonist, in transgenic mice and reported a significant decrease in bone density and bone formation in these animals [88]. In contrast, complete knockout of noggin led to irregularly thickened bones and death shortly after birth [85, 89]. Other BMP antagonists include chordin and gremlin. Multiple *in vitro* studies have demonstrated that chordin is a strong endochondral ossification stimulator [90–93]. Zhang et al. [94] examined the role of chordin *in vivo* and their results show that BMP-2 enhances maturation of chondrocytes resulting in growth of the growth plate of Hamburger-Hamilton stage 25-27 embryonic chick limbs. When chordin (a BMP antagonist) was expressed ectopically, it resulted in a delayed growth rate of the growth plate by binding to BMP to inhibit BMP's function. From previous *in vitro* studies it is known that when gremlin-1 is suppressed, the expression of osteoblastic genes ALP, BSP, MSX2, OC, OPN, and RUNX2 is significantly increased [95]. It was only recently that the role of gremlin was investigated *in vivo*. Rowan et al. [96] explored the effect of *Grem 1* deletion in *ROSA26CreER-Grem1 flx/flx* mice. Although these mice demonstrated normal bone structure, there were other abnormalities present including severe bowel disruption as well as abnormal haematopoiesis. Cyclooxygenase 2 (COX-2) enhances *in vitro* osteogenic differentiation through initiating the BMP signalling pathway via a positive regulatory loop with BMP9, a potent osteogenic stimulator [97, 98]. Wang et al. [98] demonstrated that COX-2 is critical for orchestrating the BMP/Smad signalling pathway *in vitro* (Figure 3). Silencing Cox2 downregulated the expression of RUNX2 and Dlx-5. Similarly, *in vivo* studies showed that COX-2 knockout mice displayed 98% and 86% reduction in bone formation when they received a bone graft from other COX-2 knockout mice or wild type mice, respectively [99].

Foxc1 is another important osteogenic regulator that interacts with an osteogenic factor, BMP4. Foxc1 mutant mice display numerous abnormalities related to bone development. The calvarial bones and sternum are absent, the ribs are deformed, and the skull base is reduced in size [100, 101]. The ectopic expression of Foxc1 in C2C12 myoblasts resulted in the rescue of osteogenesis by increasing ALP activity and inducing early osteogenic markers such as RUNX2 and type I collagen [102]. Furthermore, Hopkins et al. [103] demonstrated that a downregulation of Foxc1 in C2C12 cells resulted in the inhibition of RUNX2, Msx2, and ALP activity (Figure 3). These investigators suggested that Foxc1 is required for the initiation of osteogenesis but not for the later stages, as they observed a decrease in Foxc1 levels as differentiation proceeded.

### 3.6. Wnt/*β*-Catenin Signalling Pathway

Wnt/*β*-catenin signalling, also known as the classical or canonical Wnt pathway, is of particular importance as it can either induce or inhibit osteogenesis. This pathway can regulate the expression of RUNX2 to induce osteogenesis. Alternatively, the Wnt pathway inhibits osteogenesis by altering the OPG/RANKL ratio. The expression of PPAR*γ* is also controlled by the Wnt pathway. PPAR*γ* is the main transcription factor in adipogenesis, and therefore, its expression needs to be inhibited in order for osteogenesis to occur [35] (Figure 3).

Mice lacking the *Lrp5* gene, which codes for a Wnt coreceptor, developed osteopenia, while the overexpression of *Lrp5* resulted in high-bone-mass syndromes [104, 105]. Genome-wide association studies in humans revealed an association between multiple mutations in Wnt1 and Wnt16 and early onset osteogenesis imperfecta and osteoporosis; both bone disorders result in brittle bones as well as an increased risk of fractures [106, 107]. Hilton et al. [33] removed all the components of the Notch network in mice, and this resulted in increased bone mass and a depleted pool of MSCs in the bone marrow [33]. The Notch network inhibits osteogenesis through the expression of HEY1 and HEYL transcription factors that directly inhibit RUNX2 (Figure 3). Overexpression of Notch-1 in mice inhibited osteogenesis through the inhibition of the Wnt/*β*-catenin signalling pathway [108]. It is clear that extracellular signalling pathways play a major role in osteogenesis via a complex network of transcription factors. It is therefore important to examine the network as a whole and not separate out specific interactions, as would occur in an *in vitro* setting.

## 4. Mesenchymal Stromal/Stem Cells

MSCs contain a population of multipotent adult stem cells capable of differentiating into cell types of mesodermal origin [109]. MSCs were initially isolated from bone marrow (BM) and are in this setting referred to as bone marrow-derived MSCs (BM-MSC) [110]. Since then, human MSCs have been isolated from various foetal and adult tissues, such as adipose tissue [111], the amniotic membrane [112], amniotic fluid [113], placental and foetal membranes [114], umbilical cord lining membrane [115], the endometrium [116], dental tissue [117], menstrual blood [118], peripheral blood [119], skin [120], synovial fluid [121], and Wharton's jelly [122].

It is well accepted that isolated MSC populations are heterogeneous, containing both stem cells and mature stromal cells. Even though the terms mesenchymal stem cells and mesenchymal stromal cells are used interchangeably [109], there are distinct differences between the two. Mesenchymal stem cells possess the ability to self-renew and differentiate, demonstrating the functionality of true stem cells, while mesenchymal stromal cells refers to a heterogeneous populations of progenitor cells at various stages of maturation. Directly after isolation, the isolated MSC population may also contain differentiated cells present in the tissue microenvironment such as endothelial cells, pericytes, fibroblasts, and immune cells, as well as elements of circulating blood [123–125].

## 5. Characterization of MSCs

All MSCs, independent of their source, should adhere to minimal criteria recommended by the International Society for Cellular Therapy (ISCT). These include (a) the ability to adhere to plastic; (b) the expression of a specific set of cell surface markers such as cluster of differentiation (CD)73, CD90, CD105 or CD13 and the lack of CD14, CD19, CD31, CD45, and human leukocyte antigen (HLA)-DR; and (c) the ability to differentiate into at least adipocytes, osteoblasts, and chondrocytes *in vitro* [126]. Many studies have suggested that the expression of CD34 is variable and therefore MSCs can either be positive or negative for CD34 [126–128]. Currently, there is no cell surface protein specific to MSCs, and MSCs isolated from different sources may differ regarding cell surface protein expression profiles.  Table 1 summarizes the different cell surface markers that are associated with MSCs isolated from different tissue sites.

## 6. Osteogenic Potential of MSCs *In Vitro*

*In vitro*, MSCs are induced to undergo osteogenic differentiation following exposure to compounds such as *β*-glycerophosphate, dexamethasone, and ascorbate-2-phosphate, that promote cell proliferation and osteogenic differentiation. Although these 3 compounds (*β*-glycerophosphate, dexamethasone, and ascorbate-2-phosphate) are present in all *in vitro* osteogenic media, there is a lack of consensus regarding the optimal medium for *in vitro* osteogenic differentiation of MSCs, particularly regarding the concentration of dexamethasone, which varies significantly between studies.  Table 2 summarises the composition of the osteogenic media used most often. Ascorbic acid and dexamethasone are the main osteogenic inducing factors, and together increase the activity of ALP. Upregulation of ALP activity increases the speed at which bone differentiation occurs [129]. Ascorbate-2-phosphate is responsible for the synthesis of collagen in the early stages of osteogenesis, while *β*-glycerophosphate is responsible for mineralization in the later stages [130, 131]. Along with increasing ALP activity, dexamethasone also regulates the osteogenesis-related gene RUNX2 [132].

Various spectrophotometric assays are used to determine the extent of *in vitro* osteogenic differentiation. Both the Von Kossa assay and the Alizarin Red S (ARS) assay stain for calcium deposits that are present in bone tissue. The Von Kossa assay is a qualitative assay in which calcium is replaced with silver ions (source: silver nitrate solution) to form black/brown deposits that can be analysed under a microscope [133]. The ARS assay is semiquantitative in which ARS reacts with calcium to form a red deposit which is extracted using acetic acid. The extracted dye is spectrophotometrically quantified at 405 nm [134]. Another assay that is often used to quantify osteogenesis is the ALP assay that also uses spectrophotometry to measure the level of ALP activity. In short, 4-nitrophynylphosphate is used as a phosphate substrate for ALP which dephosphorylates 4-nitrophenylphosphate which then turns yellow. This colour change is measured at 405 nm [135].

MSCs isolated from various tissues also differ in their differentiation capabilities [152–155]. This may be due to DNA methylation of key transcription factors. Xu et al. [152] demonstrated that MSCs retain their epigenetic memory and favour either of adipogenic or osteogenic differentiation, depending on their tissue of origin. In BM-MSCs, the CpG island in the RUNX2 promoter is hypomethylated while the CpG island in PPAR*γ* is hypermethylated. The opposite is true in adipose tissue-derived stromal/stem cells (ASCs): the PPAR*γ* promoter is hypomethylated while the RUNX2 promoter is hypermethylated. Pérez-Silos et al. and McLeod et al. [156, 157] suggest that MSCs consist of subpopulations that share common features while varying in the expression profile of their cell surface proteins, which can be related to differences in differentiation potential. Cantentin et al. [158] found that UC-MSCs produced significantly more ECM, while stronger staining for type I collagen was observed for BM-MSCs indicating that BM-MSCs have enhanced osteogenic potential when compared to UC-MSCs. UC-MSCs produced molecules that BM-MSCs did not such as type X collagen and the HtrA1 gene product. UC-MSCs additionally displayed a higher proportion of CD73+ cells. The authors suggest that the difference in CD73 expression and the production of these atypical molecules are the major reason for differences in chondrogenic differentiation potential between BM-MSCs and UC-MSCs.

Other factors that may influence the differentiation capabilities of MSCs include the age of the donor, the health of the donor, culture conditions, and method of isolation. Barboni et al. and Xin et al. [159, 160] both demonstrated a positive correlation between age and DNA methylation status. Barboni et al. [159] observed a correlation between gestational age of amniotic-derived MSCs and global DNA methylation status, which resulted in a decrease in osteogenic differentiation potential. Xin et al. [160] extensively compared DNA methylation status and multilineage differential capabilities. An age-related decline in ASC osteogenic differentiation was observed when ASCs from young and old donors were compared. In another study, the differentiation potential of BM-MSCs from patients with osteoarthritis (OA) was compared to MSCs isolated from a control group of a similar age: both the chondrogenic and adipogenic differentiation potential of BM-MSCs from OA patients were significantly decreased compared to controls, while the osteogenic potential was similar when BM-MSCs from OA patients and MSCs from the control group were compared [161].

He et al. [174, 175] demonstrated that the extracellular matrix is important in directing MSCs down a specific lineage: a hydroxyapatite- (HA-) collagen matrix was found to be superior to a HA-synthetic hydrogel for osteogenic differentiation. For chondrogenesis, the HA-synthetic hydrogel was preferred over the HA-collagen matrix. The HA-collagen matrix imitated the natural composition of bone and resembled the physical and chemical microenvironment found in the human body, thus favouring osteogenesis. The reason why the HA-synthetic hydrogel was favoured for chondrogenesis is not fully understood, as the HA-synthetic hydrogel does not imitate natural cartilage. Overall, the use of a matrix increased cell proliferation, adhesion, migration, and differentiation. The biomechanics of the MSC microenvironment also has an effect on differentiation capabilities. Gungordo et al. [176] concluded that rat BM-MSCs progress to an adipogenic lineage under unstrained conditions on a softer polyacrylamide hydrogel film, while rat BM-MSCs seeded on a stiffer polyacrylamide hydrogel and under strained conditions are driven down the osteogenic lineage. The use of animal serum which contains xenoantigens is another culture condition that can affect differentiation potential, specifically osteogenic differentiation [177, 178]. Okajcekova et al. [179] compared three different osteogenic induction media and their differentiation capabilities, of which one was xeno-free. Not only did the xeno-free induction medium result in significantly greater osteogenic differentiation potential compared to the other two, but the morphology of the cells grown in the xeno-free medium changed much earlier than the cells grown in the FBS induction medium: cell proliferation decreased while cell differentiation increased.

The method of isolation also has an impact on the differentiation capability of MSCs. In a recent study by Walter et al. [180], different isolation techniques from the same donor site were compared with regard to osteo-, adipo-, and chondrogenic differentiation. MSCs isolated from bone marrow aspiration showed better osteogenic differentiation than MSCs generated through outgrowth from culturing bone chips, which can be attributed to the fact that bone marrow aspiration yields more biomaterial and thus more MSCs. Chondrogenic and adipogenic differentiation, both from MSCs from bone marrow aspiration and MSCs generated through outgrowth from culturing bone chips, was relatively low; the authors attribute this to the specific microenvironment of the isolated bone tissue and suggest that this led to MSCs favouring the osteogenic lineage.

Musina et al. [181] compared the osteogenic differentiation potential of MSCs from different tissue sources after a three-week induction period. These investigators reported that BM-MSCs displayed the highest level of osteogenic differentiation, followed by ASCs which showed better osteogenic differentiation capabilities than MSCs isolated from the thymus, skin, and placental tissues. Mohamed-Ahmed et al. [182] compared the osteogenic potential of MSCs isolated from bone marrow and adipose tissue and also reported that BM-MSCs possess enhanced osteogenic potential when compared to ASCs. The reason for the difference was attributed in part to increased alkaline phosphatase (ALP) activity and osteogenic gene expression kinetics. Early-stage osteogenic genes such as RUNX2, collagen type I, and ALP were expressed as early as day 14 in osteogenic differentiating BM-MSCs, while these genes were only expressed on day 21 in differentiating ASCs. This indicates that BM-MSCs stop proliferation early (day 14) and switch to differentiation and formation of a mature collagenous matrix, while ASCs have an extended proliferation period and only switch to differentiation after day 21, resulting in BM-MSCs having greater mineralization and therefore more bone tissue on day 21 [182]. Shen et al. [153] compared MSCs derived from the amniotic membrane (AM-MSCs), the umbilical cord (UC-MSCs), the chorionic membrane (CM-MSCs), and the decidua (DC-MSCs) and reported enhanced osteogenic differentiation (based on ARS staining and ALP activity) in AM-MSCs and UC-MSCs when compared to CM-MSCs and DC-MSCs. In terms of gene expression profiles involved in osteogenesis, AM-MSCs and UC-MSCs showed strongly enhanced expression of Ocn compared to CM-MSCs and DC-MSCs. MSCs from all four sources showed the same expression levels of Osx and collagen type I on day 21. Szöke et al. [183] compared the osteogenic potential of MSCs isolated from bone marrow and adipose tissue. They concluded that although ASCs had a higher proliferative capacity and a greater ability to form a collagenous extracellular matrix, their terminal osteogenic differentiation capability was reduced. BM-MSCs expressed a higher level of the late osteogenic markers Ocn and BSP. They further went on to suggest that ASCs may be more suitable for *in vitro* studies, as their isolation procedure is less invasive than BM-MSCs, and although their terminal differentiation capability is reduced, it is still adequate for *in vitro* studies, while BM-MSCs may hold greater potential for *in vivo* studies as their terminal osteogenic differentiation capability is greater than that of ASCs. For more information on the differences between BM-MSCs and ASCs with regard to their osteogenic potential, we refer the reader to a review by Liao [184].

Multiple barriers limit the clinical application of MSCs. Many of these are related to the need to extensively expand these cells *ex vivo* in order to achieve clinically relevant cell numbers. One major barrier associated with extensive *ex vivo* expansion is the decrease in differentiation potential, mainly due to the loss of telomerase activity, also known as replicative senescence [185]. MSCs tend to lose their differentiation potential as passage number increases, and it is thus important to limit expansion rounds, ideally staying below 5 passages [186, 187]. Bonab et al. [187] reported that MSCs, especially BM-MSCs, show a lower multilineage differential potential due to morphological changes and a decrease in telomere length resulting in the loss of MSC characteristics during long-term culturing. The thawing of MSCs preserved in liquid nitrogen results in a heat-shock response (“cryogenic injury”) which leads to a decrease in their immune modulatory function [188]. Another disadvantage of long-term culturing is an increase in the probability of malignant transformation, in which cells acquire cancer-like properties [189].

A further limit to *in vitro* cell culturing is the use of foetal bovine/calf serum (FBS/FCS) as a supplement to cell culture medium to ensure optimal cell proliferation [190]. Although commonly used, FBS/FCS-supplemented growth media are associated with number of disadvantages. First, FBS/FCS shows batch-to-batch variation due to the variable composition of the product, and thus, results are often not reproducible [191]. Furthermore, FBS/FCS is xenogeneic and contains bovine proteins that can potentially elicit an immune response in humans [192]. The transmission of zoonotic diseases is also a possibility and thus also a primary concern when culturing cells in FBS/FCS; cells that have been cultured in FBS/FCS can therefore not be used clinically [193].

Due to the disadvantages associated with FBS/FCS, the use of human blood products in cell culture medium as alternatives to animal serum is becoming increasingly popular [194, 195]. In short, blood is separated by centrifugation into its components, i.e., platelets, growth factors, and fibrin, which are separated from erythrocytes [196]. Some of these blood products include human serum (HS), platelet-rich plasma (PRP), platelet-poor plasma (PPP), fresh frozen plasma (FFP), and human platelet lysate (HPL). Human serum (HS) is produced by taking whole blood donated from a patient, allowing it to clot and centrifuging the blood to produce serum that is devoid of platelets, erythrocytes, and leukocytes [197]. Plasma is the noncellular liquid part of whole blood. Two human alternatives can be prepared from plasma: PRP and PPP. The difference is the concentration of platelets. Platelets are anucleated, disc-shaped cell fragments that play a role in cell growth, differentiation, and tissue regeneration [198]. When preparing PRP, whole blood is centrifuged, and the supernatant (plasma) is centrifuged again to collect a platelet pellet; the platelet pellet is then resuspended in a smaller volume of plasma thus combining the plasma and buffy coat into one [199]. Alternatively, PRP can be collected via apheresis [200]. PPP is prepared by removing platelets from the plasma obtained from whole blood. Fresh frozen plasma (FFP) is obtained by rapidly freezing plasma separated from whole blood at -65°C [201]. Lastly, to produce HPL, PRP is submitted to several freeze-thaw cycles to rupture the platelets releasing growth factors, followed by centrifugation to remove cell debris [200]. The various human alternatives provide unique advantages and disadvantages with regard to culturing MSCs in *in vitro* by providing suitable growth factors and ensuring genomic stability [197].

## 7. Bone Regeneration and Repair: Clinical Application

Bone is a vital part of the human body that protects and supports various organs, enables mobility, stores minerals, and produces cells of the hematopoietic lineage [202]. Bone fractures typically heal without the need for major intervention; however, there are more than 2 million cases worldwide in which patients require bone reconstruction using tissue transplants [203]. Current reconstruction procedures involve autologous bone grafts, allogeneic bone grafts, and artificial metal or ceramic replacements.

Autologous bone grafts are viewed as the gold standard for treating bone defects as they enhance osteogenesis and are less likely to be rejected by the host [204]. However, 10% of bone harvests are associated with major complications, limited supply, and donor-site morbidity [205, 206]. Allogeneic bone grafts provide an ample source of tissue, but the risk of immune rejection and the transmission of diseases make them less ideal [207]. The use of metals as artificial replacements also has limitations such as tissue-host integration, increased risk of infection, and wearing out [208]. The brittle nature of ceramic replacements is especially problematic in areas where high stress or torsion is endured [209]. It is thus clear that alternative, more effective options are needed for the treatment of skeletal defects.

## 8. The Use of Cultured MSCs for Osteogenesis *In Vivo*

The osteogenic differentiation potential of multipotent MSCs has gained increasing interest in tissue engineering especially when it comes to offering an alternative to overcome the limitations of bone grafts and artificial replacements [210]. Multiple *in vitro* studies have demonstrated that MSCs are able to differentiate into bone tissue, but bone formation is a complex process that involves many cell types, growth factors, cytokines, and mechanical stimulation that all form part of the environmental niche [211]. Therefore, investigation of bone formation *in vivo* is required to provide a complete understanding of osteogenesis and also bridges the gap between the use of MSCs *in vitro* and the clinical use of MSCs for bone repair.

Most studies that have investigated osteogenic differentiation of MSCs *in vivo* first expanded the cells *ex vivo*, seeded them onto a scaffold, and transplanted the scaffold subcutaneously in an animal model in which osteogenesis was studied [212–214]. For optimal bone regeneration, the biomaterial used as a scaffold should be biocompatible, cost effective, biodegradable, and should also induce or improve the osteogenic process. The biological behaviour of MSCs is greatly affected by the surface morphology of the biomaterial which in turn affects the formation of bone tissue [215]. The most common scaffold material being used in tissue engineering is hydroxyapatite, an inorganic material that is naturally found in bone tissue [216]. These scaffolds are cast into the desired shape. Another new and attractive method of making scaffolds is the use of 3-dimensional (3D) printing, as it allows for a reproducible design when it comes to pore size [217]. Once the scaffolds are transplanted, MSCs differentiate into osteoblasts and form bone tissue.

Several factors play a role in inducing MSC differentiation into bone *in vivo* including paracrine signalling pathways in the region of bone injury [218]. When bone injury occurs, perivascular stem cells induce paracrine pathways through the secretion of Wnt-related molecules that in turn activate the BMP and Wnt *β*-catenin pathways causing osteogenic differentiation [219]. Furthermore, MSCs create a microenvironment that supports new bone formation through the production of an ECM [220]. Several approaches have been investigated to enhance osteogenic differentiation *in vivo* including harvesting of ECM to coat biomaterials. The use of ECM not only improves osteogenic differentiation but also enhances MSC survival *in vivo* [221–223]. Another approach is to coat biomaterials with osteogenic inductive compounds [224–226]. MSCs can also be primed or predifferentiated down the osteogenic lineage before seeding them onto scaffolds [227–229]. Lastly, MSCs can be genetically engineered to express bone inducing genes which enhances osteogenic differentiation [230–233].

Several methods, such as histological staining, histomorphometry, immunohistochemistry, and quantitative real-time polymerase chain reaction (RT-qPCR), are used to assess the success of osteogenesis *in vivo*. Once osteogenesis has been allowed to occur in *in vivo* mouse models, the scaffolds on which the new bone tissue has formed are resected, and the degree of osteogenesis is measured. Histological staining with haematoxylin and eosin is used to nonspecifically detect newly formed bone matrix [234]. A combination of Alcian blue, haematoxylin, orange g, phloxine b, and eosin serves as a more specific histological stain for mature bone tissue [235]. Immunohistochemistry allows for the identification of specific antigens such as type I collagen, Ocn, Opn, and BMP-2 [236–238]. Lastly, RT-qPCR can be used to assess the expression of osteogenesis-associated genes such as ALP, RUNX2, BSP, Osx, Ocn, Dlx5, and BMP-2 [213, 239, 240].

Angiogenesis needs to occur for successful bone healing in large bone defects. The successful translation MSC-associated cell therapy products for the treatment of bone defects in the clinical setting must be accompanied by rapid vascularization of the implanted scaffold [241]. Vascularization results in adequate delivery of nutrients, oxygen supply, and the removal of waste products. Rapid vascularization also supports the survival of the seeded cells. To promote rapid vascularization, some studies suggest coculturing MSCs and endothelial cells, the latter for their ability to promote angiogenesis [241, 242]. Other studies suggest using a cell type that has the ability to differentiate into both bone tissue and endothelial cells to improve angiogenesis *in vivo* [213]. Brennan et al. [213] used ASCs based on the assumption that ASCs can differentiate into both endothelial cells and osteoblasts; they found that although ASCs were able to achieve both osteogenesis and angiogenesis; the degree of osteogenesis was inferior to the degree of osteogenesis achieved by BM-MSCs. These investigators then investigated coculturing BM-MSCs and ASCs and found that although there was enhanced blood vessel formation, osteogenesis was not enhanced. Brennan et al. [213] concluded that ASCs need to be osteogenically primed prior to implantation to achieve enhanced osteogenic abilities. The need to prime ASCs to undergo osteogenic differentiation was supported by various other investigators who found that without priming, ASCs fail to heal critical-size defects [243–245]. Another interesting hypothesis was that the immune system and bone formation are linked. Several studies have suggested that MSCs secrete paracrine factors that recruit immune cells to the site of injury leading to bone formation [211, 246, 247]. In order to close the gap between culturing MSCs *ex vivo* and the clinical use of MSCs for the treatment of bone defects, new methods are required to improve the efficiency of osteogenesis *in vivo* through, for example, the use growth factors, and by improving methods of cytokine delivery to the implanted scaffold.

## 9. The Use of MSC-Derived Exosomes for Osteogenesis *In Vivo*

The ability of MSCs to secrete exosomes, in addition to cytokines and growth factors, contributes to their therapeutic effect [248]. Multiple *in vivo* studies have demonstrated that very few MSCs engraft at sites of injury when administered intravenously, but rather are filtered out in the lungs; however, they still exhibit a therapeutic effect [249–253]. Other studies have gone on to report that it is in particular the microvesicles/exosomes secreted from MSCs than provide this therapeutic effect [127, 254, 255]. The therapeutic effect of MSC-derived exosomes has been extensively studied *in vivo* in a wide range of disease models. Some of these include cardiovascular disease [256–258], renal disease [259–261], neurological complications [262–264], pulmonary disease [265–267], wound healing [268, 269], muscle regeneration [270], and many more. With regard to osteogenesis, multiple studies have shown that MSC-derived exosomes can stimulate the osteogenic differentiation process, increasing bone regeneration. Qi et al. [271] demonstrated that exosomes from BM-MSCs from ovariectomized rats stimulated osteogenesis and were able to regenerate bone tissue in a critical-sized calvarial defect. They also found that the increase in osteogenic stimulation was related to the increase in exosome concentration over time. The repair of a critical osteochondral defect in adult immunocompromised rats through the intravenous injection of human embryonic MSC-derived exosomes was demonstrated by Zhang et al. [272]. The use of MSC-derived exosomes in regenerative medicine has gained a great deal of attention as it is an attractive alternative to using MSCs. MSC-derived exosomes are cell-free and are more compatible with a variety of administration routes [272]. Another reason why exosomes are attractive is that they lack major histocompatibility complex (MHC) I/II proteins, and there is therefore no need for immunosuppression [273, 274].

## 10. Therapeutic Use of MSCs for Bone Diseases

Bone remodelling is a complex and highly integrated process, and as described in this review, it involves various transcription factors and osteogenic genes and their protein products including cytokines, growth factors, and extracellular matrix components. The smallest deviations from this well-balanced system can affect bone health and lead to a number of bone diseases. Bone tissue is a porous, mesh-like network made up of collagen proteins and calcium phosphate minerals and is constantly being replaced throughout life. When the bone remodelling process is defective, this mesh-like structure becomes porous as seen in osteoporosis, leading to brittle bones and fractures.

According to the International Osteoporosis Foundation, over 200 million people are affected by osteoporosis worldwide [275]. Osteoporosis is associated with low bone mass as well as bone deterioration usually seen with increasing age, and it is thought that osteoporosis results, in part, from a significant decrease in the number of MSCs present in the bone marrow, leading to less new bone formation [276]. Osteoporosis is currently treated with drugs that increase bone resorption, but these drugs are associated with multiple adverse effects [277]. Stem cell therapy is a potential alternative for the treatment of osteoporosis, reducing the susceptibility to fractures by increasing the MSC pool present within the bone marrow. Wang et al. [278] reported increased bone formation, trabecular thickness, and overall strength of bone tissue by embedding MSCs into the distal femurs of osteoporotic rabbits. Hsiao et al. [279] treated osteoporotic mice by injecting MSCs intravenously. They observed that the MSCs homed to the bone marrow where they increased bone density, rescuing the mice from osteoporosis.

OA is a degenerative joint disease affecting synovial joints and frequently results in chronic pain [280]. Currently, the treatment of OA involves long-term pain management with the use of pharmacological therapies. Osteotomy can improve alignment, but this therapy is limited as it can decrease the risk of OA but has little effect on degeneration once it has occurred [281]. It has been hypothesized that the multipotency properties of MSCs could also benefit patients with OA. Currently, there are 102 documented clinical trials assessing the potential of MSCs for the treatment of OA; however, more preclinical work is needed to fully understand the mechanisms behind the potential healing effect of MSCs in OA [280]. Eder et al. [282] comprehensively reviewed the use of both ASCs and BM-MSCs in the treatment of musculoskeletal disorders, including OA. The overall conclusion of these studies is that the use of MSCs (ASCs and BM-MSCs) decreases pain levels and improves healing rates. Eder et al. [282] concluded that MSCs could be used as a therapeutic option in the future treatment of OA, although the field would benefit from large, randomized, blinded clinical investigations.

The repair and reconstruction of large segments of bone, such as fractures displaying nonunion or delayed union, and large bone defects, have been a challenge to orthopaedic surgery. MSCs are an attractive therapeutic option for the healing of bone defects due to their ability *in vivo* to influence the secretion of specific factors by the immune system and through their interaction with other cells [283]. A number of clinical trials have been conducted with the aim of assessing the ability of MSCs to improve fracture healing. In most cases, improved fracture healing rates, decreased pain levels, and improved remodelling have been observed when compared to controls [284–291]. The conclusions that can be drawn from these studies indicate that the use of MSCs could be an important treatment option for larger more difficult bone defects in the future. Many more bone diseases could benefit from bone regeneration therapy and hence the importance of understanding the osteogenic process in full.

## 11. Conclusions

The multipotent differentiation and secretory capabilities of MSCs makes them attractive for transplantation and regenerative therapies particularly in treating bone defects/disorders. MSCs can successfully differentiate into osteoblasts *in vitro*, and multiple studies in this setting have provided methods to improve the differentiation process. MSCs can also be transduced effectively to express genes of interest that can further improve osteogenesis *in vitro*, and it is likely that this will be maintained *in vivo*. In an *in vivo* setting, cultured MSCs retain their ability to differentiate into osteoblasts. The transcriptional regulation of osteogenesis has not been fully elucidated. Further understanding of bone biology will rely on deciphering the complex regulatory network and multiple interactions between regulatory factors. The lack of cell-culturing systems that fully replicate osteogenic differentiation *in vivo* has made the understanding of transcriptional regulators that control osteogenesis difficult. It is however important to ensure the safe and effective use of MSCs in the therapeutic setting. Therefore, more *in vivo* studies are needed to address the numerous *in vitro* discrepancies. The use of MSCs in the clinical setting awaits final validation, but there are multiple ongoing clinical trials that show promising results. Fundamental questions regarding the biology of osteogenesis remain, and the therapeutic potential of MSCs needs to be fully explored before they can be used as a routine treatment option. The field is nonetheless highly promising, and important contributions to the practice of medicine can be expected.

## Figures and Tables

**Table 1:** Cell surface markers expressed by MSCs isolated from different tissues.

Source	Cell surface marker		Reference
	Positive	Negative	
Adipose tissue	CD10, CD13, CD29, CD34, CD44, CD49e, CD59, CD71, CD73, CD90, CD105, CD166, CD200, HLA-ABC	CD11b, CD14, CD19, CD31, CD34, CD45, CD56, CD146, CD235a, Stro1, HLA-DR	[111, 136–139]
Amniotic membrane and fluid	CD29, CD44, CD73, CD90, CD105, SH2-4 HLA-ABC	CD11b, CD10, CD14, CD19, CD20, CD34, CD45, CD79a, HLA-DR	[113, 140–142]
Bone marrow	CD29, CD44, CD73, CD90, CD105, CD271, Stro-1	CD14, CD34, CD45, HLA-DR	[57, 126, 137, 143]
Dental tissue	CD29, CD34, CD44, CD73, CD90, CD105, CD105, CD117, CD166, Stro1	CD11b, CD14, CD19, CD31, CD34, CD45, CD79a, CD146, HLA-DR	[117, 143–145]
Endometrium	CD44, CD49d, CD479f, CD73, CD90, CD105, CD146	CD14, CD19, CD34, CD45, HLA-DR	[116, 146]
Peripheral blood	CD29, CD73, CD90, CD105, CD106, CD146, CD166,	CD34, CD45, CD133	[119, 147, 148]
Placental and foetal membrane	CD29, CD73, CD90, CD105	CD34, CD45	[114]
Skin	CD29, CD44, CD73, CD90, CD105, CD166	CD14, CD34, CD45, HLA-DR	[120, 149]
Synovial fluid	CD44, CD73, CD90, CD105, CD147, Stro-1	CD11b, CD14, CD19, CD31, CD34, CD45, CD79a, CD106, HLA-DR	[121, 141]
Umbilical cord lining membrane	CD29, CD44, CD73, CD90, CD105, CD106, HLA-I	CD14, CD31, CD34, CD45, HLA-DR	[111, 115, 150]
Wharton's jelly within umbilical cord	CD73, CD90, CD105	CD14, CD19, CD34, CD45, CD79, HLA-DR	[122, 151]

**Table 2:** Summary of different osteogenic differentiation media reported in the literature.

Reference	Cell density	Assays/stains	Passage	Induction time (days)	Basal culture medium	FBS	Antibiotics	Dexamethasone (*μ*M)	Ascorbate-2-phosphate (*μ*M)	B-glycero-phosphate (mM)
Cai et al. 2014 [162]	NI	Alizarin Red S and ALP	2	21	DMEM-low glucose (lg)	10%	100 units/mL	0.01	155.26	1 × 10^−5^
Vieira et al. 2010 [163]	NI	Von Kossa	3	21	NI	10%	NI	0.1	50	1 × 10^−5^
Nishimura et al. 2015 [129]	5 × 10^5^		5	14	DMEM	10%	100 units/mL	0.05	0.0002	10
Bieback et al. 2004 [164]	3.1 × 10^3^/cm^2^	Von Kossa	NI	21	Cell systems	10%	NI	0.1	50	10
Waterman et al. 2010 [165]	3 × 10^4^ cells/well (6-well)	Alizarin Red S	NI	NI	NI	NI	NI	0.1	50	1 × 10^−5^
Elashrya et al. 2019 [166]	2 × 10^4^ cells/well (6-well)	Alizarin Red S	2-3	NI	DMEM	10%	100 U/mL	0.1	60	10
Li et al. 2015 [167]	5 000 cm^2^	ALP	NI	NI	DMEM-high glucose	10%	100 U/mL	0.01	155.26	10
Sotiropoulou et al. 2006 [168]	NI	Von Kossa	NI	NI	DMEM-lg	10%	50 *μ*g/mL Gentamicin	1	50	10
Rada et al. 2011 [169]	NI	Alizarin Red S	NI	21	*α*-MEM	10%	1%	0.1	155.26	10
Meuleman et al. 2006 [170]	NI	Von Kossa	1	14	*α*-MEM	NI	NI	0.1	60	10
Sasaki et al. 2008 [171]	NI	Von Kossa	NI	NI	DMEM	10%	0.1 *μ*M	-	50	10
Zuk et al. 2001 [172]	NI	ALP or Von Kossa	1	14	DMEM	10%	1%	1	50	10
Bunnell et al. 2008 [173]	NI	Alizarin Red S	NI	14	*α*-MEM	20%	1%	0.001	50	2
Wagner et al. 2005 [111]	1 − 2 × 10^4^ cells/cm^2^	ALP or Von Kossa	NI	21	DMEM	10%	-	1	200	10

NI: not indicated; DMEM: Dulbecco's modified Eagle's medium; ALP: alkaline phosphatase.
